# Integrating the results of user research into medical device development: insights from a case study

**DOI:** 10.1186/1472-6947-12-74

**Published:** 2012-07-19

**Authors:** Jennifer L Martin, Julie Barnett

**Affiliations:** 1 Department of Electrical and Electronic Engineering, Faculty of Engineering, University of Nottingham, Nottingham, NG7 2RD, UK; 2 School of Information Systems, Computing & Mathematics, Brunel University, Uxbridge, Middlesex, UB8 3PH, UK

**Keywords:** Medical device, Product development, User centred-design

## Abstract

**Background:**

It is well established that considering users is an important aspect of medical device development. However it is also well established that there are numerous barriers to successfully conducting user research and integrating the results into product development. It is not sufficient to simply conduct user research, it must also be effectively integrated into product development.

**Methods:**

A case study of the development of a new medical imaging device was conducted to examine in detail how users were involved in a medical device development project. Two user research studies were conducted: a requirements elicitation interview study and an early prototype evaluation using contextual inquiry. A descriptive *in situ* approach was taken to investigate how these studies contributed to the product development process and how the results of this work influenced the development of the technology. Data was collected qualitatively through interviews with the development team, participant observation at development meetings and document analysis. The focus was on investigating the barriers that exist to prevent user data from being integrated into product development.

**Results:**

A number of individual, organisational and system barriers were identified that functioned to prevent the results of the user research being fully integrated into development. The user and technological aspects of development were seen as separate work streams during development. The expectations of the developers were that user research would collect requirements for the appearance of the device, rather than challenge its fundamental concept. The manner that the user data was communicated to the development team was not effective in conveying the significance or breadth of the findings.

**Conclusion:**

There are a range of informal and formal organisational processes that can affect the uptake of user data during medical device development. Adopting formal decision making processes may assist manufacturers to take a more integrated and reflective approach to development, which should result in improved business decisions and a higher quality end product.

## Background

Whilst it is well established in theory that considering users and their requirements is an important aspect of medical device development, in practice there is little evidence of how this is done, how the results of this are incorporated into the product development process, or indeed of the difficulties of doing both of these things. Buckle et al. [1] makes the point that more case studies that document the whole spectrum of success and failure in terms of integrating the results of user needs research are essential. As a preface to presenting a case study of user involvement in medical device development and an analysis of the barriers to incorporating the resultant knowledge in the device development process, we will outline the imperatives to user involvement and provide a brief overview of the barriers to achieving this.

It is well accepted that medical devices should be designed to fulfil the requirements, needs and capabilities of their users. User involvement in medical device development has been the focus of much recent research [2-4]. The benefits of a user-centred approach to design include improved patient safety [5], better health outcomes [6] and increased user satisfaction [7]. It has also been suggested that products that have been designed in this way are more likely to be commercially successful and less likely to require post-market alterations or recalls [8]. To increase the likelihood of producing such a device, developers must have a clear and thorough understanding of, as appropriate, the clinicians, patients and carers who will use the device. The range of physical and organisational environments within which device use will be embedded must also be considered.

There are three main factors that encourage and motivate developers to conduct user research during the medical device development process (MDDP). The most notable is the regulatory imperative; there are a range of requirements that must be met before a device can legally be placed on the market. Both the US Food and Drug Administration (FDA) and the European Union require developers to demonstrate, normally through compliance with standards, that they have adopted human factors engineering processes (also known as human factors, ergonomics or user-centred design) throughout development [9,10].

A second imperative is located in the requirements of funders of healthcare technology research, whose funding decisions are, in part at least, contingent on developers promising to consult and actively involve device ‘users’ and ‘stakeholders’. This is particularly relevant for Small and Medium Sized Enterprises (SMEs) who are often dependant on this type of funding to support new product development. In the UK in particular a significant proportion of medical device development is undertaken by SMEs, for example small engineering firms and university spin-outs. These types of companies will typically have less access to in-house expertise in user research and are therefore likely to be especially reliant on securing external funding to allow them to conduct this work.

Thirdly, there is a substantial literature on methods for accessing user needs that is, in theory at least, available to device developers who wish to respond to these imperatives and conduct user centred research as part of the MDDP. This literature describes the theory behind this approach [11]; how and when to conduct the work, and with what groups of people [2,3,12].

It is also well-established, however, that there are barriers to the involvement of users in this process. Drawing on other areas of product development, it is clear that some barriers are located in relation to the user. Van der Panne et al. [13] note that users have limited ability to envision novelty and thus involvement is likely to be biased toward imitative products and incremental innovation. Other barriers are located at the organisational level. Developers may limit user engagement due to resource constraints [3] or the belief that the investment required to conduct user research is not an effective use of limited resources [14]. The way in which user needs data is delivered to the organisation may not dovetail with their decision-making process about the product [15,16]. Indeed the perspective of the user provided by formal methods may not chime with the representations of the user held by developers themselves [17]. In sum, such factors may limit the extent to which user information is appropriated within the product development process.

Any new product development project is a complex process, regardless of the industry it is located in. In recognition of this, considerable research has been conducted on how to effectively manage product development projects, and how to use the information collected during the project to make good business decisions. The question here is, how can user data be effectively collected, represented and utilised during the product development process?

Empirical research has consistently shown that that the factor most strongly associated with successful product development is “the existence of a high-quality, rigorous new product process: one that emphasizes up-front homework, tough Go/Kill decision points, sharp early product definition, and flexibility” [18]. The ‘stage-gate’ model is the most frequently used such approach [19] and aims to take the product from concept all the way to market. At the beginning of a project the development process is divided into ‘stages’ that consist of related and parallel activities. At the end of each of these stages there is a decision point or ‘gate’, when the management team reviews progress across all the different strands of development and then takes a decision on how the project should progress, or indeed, whether it should progress. The ultimate aim of such an approach is to ensure that objective and informed decisions are made at each and every stage of product development, based on careful consideration of all the information available at that time. It has recently been suggested by Pietzsch et al. [20] that the complex nature of medical device development necessitates a formal stage-gate approach to development as this industry is characterised by high technology development, strict regulatory requirements and complex reimbursement processes.

In summary then, although human factors engineering methods are potentially of value at every stage of the MDDD process and documenting user involvement is increasingly mandatory, in practice, manufacturers may have good reasons not to extensively involve users in the MDDP process. If the full benefits of involving users are to be realised, more primary research is needed to better understand manufacturers’ perspectives and motivations and also how the results of user research can be effectively incorporated into the product development process.

This paper presents a case study of a medical device development project. The results are used to recommend strategies for effective user involvement in medical device development. We also examine the wider implications of these findings for medical device manufacturers, research funders and policy makers.

## Methods

### Case study

The development of a new medical imaging device was studied using a descriptive approach, as described by Yin [21]. The objective was to study *in situ* a process of user involvement in medical device development in order to explore how user facing work was done across the product development process; characterise the approach, attitude and expectations of the developers; and identify barriers to the implementation of the research results.

### Data collection and analysis

Data in the case study were collected by a variety of methods:

· Semi-structured interviews were conducted with members of the development team at various points throughout development.

· Participant observation at development meetings.

· Document analysis of (for example) meeting minutes, project plans and technical reports.

### Ethical considerations

The research described in this paper received ethical approval from the NHS Nottingham Research Ethics Committee 2 (ref 09/H0408/15).

### Background to case study

In 2005 a development team was awarded a grant of approximately £1 M from the UK Technology Strategy Board (formerly the Department of Trade and Industry) to develop a new medical imaging device. The original project plan was that development would run for 36 months, however, due to delays, this was extended to 48 months. The project team consisted of 9 individuals of different specialities from 5 organisations. The project was led by the managing director of the lead organisation: a SME medical device developer, with a track record in biomedical instruments. The device was one of a small number of development projects within the company. At the end of the development project, the lead organisation would be responsible for commercialising the device (with appropriate licensing contracts with the other participating organisations).

### The device

For reasons of commercial confidentiality it is not possible to describe the technological details of the medical device developed in the study. In brief however, the device uses laser Doppler technology to produce images of various body areas, with the aim of providing an inexpensive, portable device that will enable healthcare professionals to perform common procedures more successfully and efficiently and with less pain and discomfort to patients.

### Project plan

The original project plan consisted of five ‘work packages’:

· WP1: Development of initial imaging system (lab-based) Months 1 – 6

· WP2: Laser developments (lab-based) Months 1–16

· WP3: Optimisation of algorithms through testing technology on volunteer patients. Months 1 – 6

· WP4: Development of imaging system. Months 6–30

· WP5: Evaluation of imaging system: evaluate technological performance on a range of patients; obtain user feedback and assessment of device. Months 22–36.

The first ‘active’ participation of users was planned during WP5 with passive involvement of patients planned during WP3. In the grant application the developers stated that they intended to involve clinical users in development:

"“*Data will be collected from the hospitals and there will be consultations with nurses, phlebotomists and doctors in the evaluation process (WP5)”.*"

The project plan identified a number of interdependencies between the work packages however these were between the technological aspects. No areas of development were identified as being contingent on user feedback about the device.

In month 2 of the project the lead author (JM) joined the development team to provide expertise on conducting user research. On inspection of the project plan JM recommended that a more formal and rigorous approach to user research should be taken that would allow functional and user requirements to be collected. As a result of this, an additional work package was added. The aim of WP0 was to “Elicit user requirements via interviews with medical staff”. This was thus the first user-related work to be conducted. Again, no formal links or dependencies from this WP for future work were identified.

We now briefly describe the two user research studies in WP0 and WP5. For a full description of these investigations please refer to Martin et al. [22] and Martin et al. [23].

## Results of User Research

### Requirements elicitation (WP0)

The aims of the first phase of user research were developed by JM following discussions with the project team. The developers stated that the main objective of this work should be to collect the opinions of healthcare staff that would use the device on its design: how it should look, what size and shape it should be and how it should display data. The project team also stated they were interested in information about the work patterns of healthcare staff. JM used these discussions as well as principles of user-centred design [24] to produce the following objectives:

· Identify the clinical need for the new device and identify target clinical and patient users

· Identify any potential barriers to safe and effective use of the device.

· Collect user opinions on the design of the new device

When deciding on the focus of this study, it was acknowledged that for any medical device there are likely to be many different types of users, and that all of these groups should be considered during development. For this device, the ‘users’ will include not only the operators of the device but also the patients who will be treated by the device. In addition, the requirements of the people that will be responsible for maintaining, cleaning and setting up devices should also be considered. As the aims of this initial study were to identify the clinical need for the device and investigate its effectiveness and usability in the clinical environment it was decided that clinical staff should be the focus as they would be the potential device operators. In addition, as the primary aim of the research was to identify the patients most likely to benefit from the device, it was felt that clinicians would be an appropriate proxy for patient views at this point in development as they would be able to draw on their experiences of treating a wide range of patients from a variety of clinical areas.

All potential clinical users of the device were identified during a brain-storming session with the development team. An interview study was then undertaken with a purposive sample of representatives of each of the identified user groups: a range of healthcare professionals from the following clinical departments: renal, oncology, intensive care, neonatal, clinical research and phlebotomy.

47 individual, semi-structured, face-to-face interviews were conducted at 2 UK NHS hospitals. Each interview lasted between 23 and 44 minutes and covered:

· Problems that clinical users encountered, their causes and consequences

· The perceived need for the proposed device

· The clinical environment within which the device would be used (physical and organisational).

Data analysis was performed by JM and was organised according to the 3 main aims of the study: clinical need; barriers to adoption; design features. A written report (39 pages with 3 page executive summary) was circulated to the project team in Month 16 of the project and the main findings were presented to the team during the next scheduled development meeting (Month 17).

The most significant finding of the requirements elicitation study was in relation to the clinical need for the proposed device. The responses of the clinical users strongly suggested that the concept for the device should be changed in two main ways. Firstly in relation to the target users for the device, the research suggested that the original concept over-estimated the clinical need for the device from the phlebotomy patients. The interviews in the neonatal, clinical research and intensive care departments revealed that there was not a significant need for the device, although the staff did report that there may be some applications where it could be of some use. However, a number of previously unknown clinical needs in renal care and oncology were identified, suggesting that these areas should be the focus of device development. Second, in relation to the characteristics of the device, the original concept had stated that an important priority was to provide a device that was light and highly portable. However, the interviews suggested that it would be the time required to set-up the device and obtain an image that would most significantly affect uptake and effective use by clinicians.

The feedback from the development team following the initial presentation of the results was positive. They noted the insights that had been gained and there was ostensible agreement about the implications of these for the development process.

"*“After the interviews it seems that that application is quite limited so the fact that it identified different markets is a big positive part (of the research)”.* Team member 2"

"*“They (results) show that speed is going to be the key thing and that’s a new challenge that we didn’t know about before. That’s going to have to be a priority of the hardware and software development.”* Team member 2"

Despite this, development of the device continued according to the original project plan. No formal opportunities were created for discussing the implications these findings might have for the development of the technology. When discussed at later project meetings, the informal responses to the interview data was that it would be considered more fully within the commercialisation plan at the end of the project once the technology had been developed.

### Prototype evaluation (WP5)

The second user research study was conducted, according to the initial plans, as part of the prototype device evaluation in WP5. The protocol was developed by JM following discussions with the development team. Based on well-established user-centred design principles the broad objective to ‘obtain user feedback and assessment of device’ [24] was extended to critically evaluate the prototype imager within the clinical environment to investigate:

· The usability of the device in the clinical environment

· Environmental and organisational barriers to safe and effective use of the device

· Opinions of users on the design of the device and the display of data

This study used an adapted form of contextual inquiry [25], an observational research method with its roots in ethnography - to study a total of 12 users (healthcare staff) as they used the device during patient consultations. The researcher (JM) shadowed each user as they took images from the patient and encouraged them to describe what they were doing and why, and asking for clarification where required. Users were encouraged to describe their interactions with the device: for example how useful they found it and whether they were experiencing any difficulties or frustrations.

This research identified a number of factors relating to the physical and organisational characteristics of the environment and characteristics and capabilities of clinical users and patients that would affect the use of the device. It was clear that the physical environment had a significant effect on the usability of the device. The device was used in a variety of areas, including individual consulting rooms of varying dimensions, and healthcare staff were observed having clear difficulties when manoeuvring the device in the smaller consulting rooms and in the presence of other medical equipment. Many patients had mobility problems or clinical conditions that made it difficult or uncomfortable for them to get into the position required to allow an image to be taken. It was also evident from the study that operational and clinical requirements would affect uptake of the device; it was a clear priority for the clinical participants to complete the patient consultations as quickly and efficiently as possible. Many expressed frustration with the length of time it took to set up and manoeuvre the device before an image could be taken. Patients also frequently expressed their desire to be treated quickly so that they could go home also suggesting that the time taken to operate the new device would affect its uptake.

Due to other work packages running over schedule a working prototype of the new device was not available for testing until the final months of the project. This meant that that the prototype evaluation did finish until the final (48^th^) month of the project and therefore was no opportunity for the researcher to formally present the full results. Following the end of the project, the development team requested the images taken from the patients during the evaluation for inclusion in the final report to the research funders and for their own records. The other findings from the evaluation were not requested and therefore the information on usability and clinical and operational requirements was not reported to the project team. No presentation of the results –formal or informal - was invited, which suggests that the developers did not perceive this information as important or likely to affect the decisions made about the future development of the technology.

### Summary of user research

The results from the user research performed were clear and consistent: there was a clinical need for the proposed device although this need was substantially different to the one that had been identified at the beginning of the project. The researcher recommended that the concept for the device should be changed to focus on the clinical need of renal and oncology patients and the requirements of the healthcare staff working in these areas. In addition, the prototype evaluation identified a number of factors related to the physical and operational characteristics of the clinical environment that would affect uptake and long-term use of the device, most notably, the time required to obtain an image.

The results of the user research were fed back to the development team in a number of ways. Following the requiring elicitation study the development team was sent a detailed written report which included a breakdown of the results according to a range of factors including: job role, clinical speciality, size of organisation and patient demographics. This was complemented by a presentation of the main findings to the whole development team. The researcher engaged informally with the development team throughout the whole project, updating the lead organisation on the progress and initial findings of the user research via e-mail. In addition, the researcher attended the development meetings (held every 3 months), where she updated the team on the findings of the user research and made recommendations for development based on the findings of this work.

The recommendations from the user research studies did not have a significant impact on the development process. Whilst the company ostensibly agreed with the results of the user needs research, the development team were happy for product development to continue in line with the original plan and thus the focus was clearly on developing the imaging technology and improving the reliability and quality of the images. The feedback from the team was that the user research data would be useful in the later stages of development.

The full results of the prototype evaluation were not reported formally to the development team, which was largely due to the fact that the study was delayed and the results were not available until the end of the project. It is interesting to note however that, although the lead organisation requested the device images that had been collected during this study, they did not also request the results of the usability testing. This suggests that the developers did not view this information as being factors that could potentially influence the decisions made about the future of the technology.

### Project outcome

Following completion of the 48 month project the lead organisation is currently considering the next step for the new medical device. The most appropriate route for commercialisation is being considered: this may be to continue to produce a clinical device or it could be to focus on producing a device for clinical research, which would be a simpler, but less lucrative, route to commercialisation. New funding and investment is being sought to support the next stages of development.

## Discussion

Our case study has focused on the user research that ran alongside the development of an imaging device and has considered the way in which the results of this were taken into account by the company. The final stages of this development project are still to unfold and therefore we are not able to draw firm conclusions as to whether these issues were a significant factor in the success or failure of this device. However the focus of this paper has been one exploring the way in which user needs research was or was not integrated into the product development process and this focus is distinct from the issue of whether or not the product ultimately proves to be a success.

It is difficult to draw any firm conclusions as to whether greater consideration of the results of the user research would have benefitted this project. However, a number of the user requirements that were identified in the early user research were at variance with the direction and focus of technological development. For example, it was determined at the beginning of the project that the device should be portable so that it could be carried around by healthcare staff; the phrase “camcorder sized” was frequently used by the development team. As a result a main focus of the technological development was on developing a device that was light and small. However, the interview data suggested that manoeuvrability at the point of capturing the image was more important than portability. The clinical users reported that getting the device to patient’s side would not be a the problem, as organisational protocols meant that it would be likely that the device would stay in a particular clinic or department, but that once the device was next to the patient it would be essential that the device could be easily positioned to quickly capture the image of the required body area. This suggests that the effort and investment that was directed to the considerable technological challenge of producing a small device may have been a poor use of resources.

There was no evidence that the user research had a significant effect on the subsequent product development process. Our analysis of this case study suggests that a range of factors can be identified as contributing to this situation.

### Integration of user needs with technology development

It was clear that the user needs part of the product development process was viewed as an isolated part of the project and separate to the technology development. The evidence provided by the requirements elicitation study was not integrated with the technology development process. This was also observed at the end of the project, when the developers requested the technical results of the prototype evaluation but not the results of the usability evaluation.

This chimes with the work of Chess [26] who provides a useful framework for exploring organisational responsiveness. She conceptualises the possible impact of organisational structure and processes on the way in which knowledge is exchanged within an organisation – and thus on the impact that this knowledge has on organisational actions. A key determinant of this is how different organisational functions are ‘coupled’, i.e. the way in which one function constrains another. In the product development scenario described in the case study it is clear that the user-facing functions (or at least those that are formally oriented around user needs) were loosely coupled to those that were related to development of the technology. The two different functions did not constrain the other, with each able to proceed independently of the other. This was evidenced by the lack of planned interdependencies between the user research and the rest of development. This is exemplified by the fact that at the end of the project, the technical results of the prototype evaluation were requested but not the usability results, suggesting that the developers did not believe that the usability results would affect future decisions about the development of the technology. There are a number of reasons why this type of situation may exist during the product development cycle. Within large organisations different aspects of development will often be performed by dedicated and separate departments and if there is poor communication or a lack of interaction between departments this could result in different aspects being de-coupled. Within a small company or a close-knit project team, such as the one in the case study, this type of situation should be less likely to occur as there should theoretically be greater involvement and awareness by all of the developing research results. However, although this was the case in this case study, there seemed to be consensus within the development team that the user needs research was not a critical aspect of development and that the results of this work would not significantly influence the development of the technology. As a result no contingencies in relation to the user needs work were identified.

We suggest that adopting a formal new product process to monitor progress and aid decision making may have led to improved integration of the different strands of this development project. The use of such processes has consistently been shown to be associated with successful product development across a variety of industries [27]. It is noteworthy that the funders of this research – the UK TSB, who support innovation in UK business by investing more than £500 million each year – did not either require or recommend that the project team adopt such a strategy for ensuring due consideration is given to the results of the user related research that *is* required.

### Framing research aims

Arguably the way in which the aims of the user research were framed by the development team was inappropriate, both in relation to the characteristics of users and the timing of the research.

### Characteristics of users

In the original product development plan no early stage of capturing clinical needs was planned. Although the developers were open to this when it was suggested, they believed that the main aim of user research was to identify design features for the new device to inform the development of a prototype. This was thus the primary aim of the requirements elicitation. The results of the case study demonstrate that this was a somewhat unrealistic aim. The data collected from the interviews indicated that users primarily expressed their reflections on the device in terms of the *needs, difficulties and problems* that they experienced. These findings support previous research that has shown that users find it difficult to provide design suggestions, particularly when unprompted by an existing prototype, as this requires a high degree of foresight [28,29]. It has also been shown that this is a particular issue for radical innovations when users do not have knowledge or experience of a similar existing product to draw on [30], which was the case with this study. Unsurprisingly, in reflecting on design features, users are also likely to draw heavily upon the contexts in which would they be required to use the device.

From this, we propose that the strategy of developers at the early stages of medical device development should be on identifying clear clinical needs and requirements. Once this has been done it is then the role of the developers and designers to provide a device that provide solutions to these needs, which can then be tested and evaluated by users and hopefully provide the required triggers for a discussion of potential improvements and refinements. In reality of course, many devices are the outcome of a new technological capability and a matching clinical need then needs to be found to allow exploitation of this. In these circumstances it is arguably all the more important that formal processes that allow the integration of revealed clinical needs with technological development are adopted.

### Timing of research

The user research commissioned by the development team did meet one of the main recommendations of the guidance and literature in this area: that research should be conducted early in the development process [31]. However our results suggest that this was negated by the lack of mechanisms for feeding the data into a product development process that was dominated by technological requirements.

The results of the requirements elicitation suggested that the fundamental concept for the device should be changed. The nature and scope of these findings was not anticipated by the developers. They rather believed that the results would confirm their belief on the clinical need for the device and provide a consensus on what the look and feel of the device should be. This suggests that it would be valuable for those doing user focused research to be clear about the potential range and scope of findings (for example, that the clinical need is different than anticipated, that unanticipated features of the device are of importance etc.). By the same token, developers need to consider and articulate what their latitude is for taking account of such findings within the product development process. Clearly it is naive to expect that all findings can be taken on board. Where time or money preclude a change in direction however, it is arguably important to document how and why this decision has been made as part of the formal stage gate process so that information is made available throughout and decisions revisited if necessary. Such a process also allows possible risks to commercial success to be identified and documented so that they can be monitored as development continues.

In our case study it appeared that neither the organisational culture nor the project management processes facilitated full consideration of the implications of the research results. That the developers did not expect the results to significantly contradict their beliefs was possibly due to their lack of familiarity with this type of research as well as to the researcher not making the potential impact of the research on the development plan clear. The researcher did not explicitly describe the scope and implications of the work, nor pose the question as to what they would do if the results were not in line with their expectations.

Even where developers are not aware of the potential benefits of user needs research a formal stage gate process would provide the opportunity for objective review of the research and clear documentation of the decisions that have been made on the basis of it. In this domain, where the assumption is that technological development has primacy, such a process provides a formal opportunity to articulate whether the user needs research that has been conducted requires any subsequent change in the development pathway.

### Significance of user research results

Another possible reason for the user research results not being fully adopted by the development team may have been the degree to which the findings contradicted the concept for the device. The concept, developed as the result of informal discussions with a small number of clinicians, had been the basis for planning the entire project, including securing the funding and as a result it is reasonable to assume that the developers were committed to this concept. It is possible therefore that cognitive biases, specifically confirmation bias, may have affected how the development team processed the results of the user research, which contradicted this concept. Confirmation bias, identified by Nickerson [32] as the “single problematic aspect of human reasoning that deserves attention above all others”, is a well-established phenomenon where decision makers have been shown to search for or interpret information in a way that confirms their hypothesis, and place less importance or ignore evidence that would contradict it [33]. This may also explain why the developers did not request the complete results of the second user research study.

Another cognitive bias that may also have affected how the results were processed is Status Quo Bias [34]: the tendency to maintain the status quo when faced with a complex decision. Recent research on this phenomena has shown that the more difficult the decision, the more likely it is that the decision-maker will do nothing and maintain the status quo [35].

Again, adopting a formal new product process may be a strategy to counter this, resulting in a more objective approach being taken to the synthesis and uptake of research evidence. This is arguably even more pertinent for SME developers where there are likely to be fewer people involved in the development of each device perhaps leading to less objectivity during decision making.

### Communicating research results

The way that the results of the first user research study were disseminated to the development team by the researcher (JM) may not have been effective in conveying the significance or breadth of the data. The full results were contained in a lengthy document that would have been time consuming for the development team to read and may also have led to the quotes losing some of their significance due to being presented as text. Playing the audio clips of these quotes may have been a more effective at conveying this information. In addition, although the most significant findings of the requirements elicitation were presented in a presentation to the development team by JM, this was done during a normal project meeting where it was one item on a full agenda. It may have been more effective to have scheduled a special meeting where the development team had more time to listen to and discuss the results.

Related to this issue is the fact that the user research was performed by an external researcher who was responsible for disseminating the information to the development team. It has been suggested that such an approach is undesirable as it inevitably results in some filtering and distortion of the information [36,37] and that developers should be brought into direct contact with users rather than merely hearing or reading about them through intermediaries [38]. In addition to this, the risk and rewards of commercialising this device lay mainly with the lead organisation and therefore it is possible that the external researcher was not sufficiently motivated to reinforce the results of this work and to press for their prioritisation during development. It is possible that this type of situation may arise in other development projects when contracting user research (or other aspects of development) to external consultancies. It is the responsibility of the developer to establish processes that will allow all information streams to be heard and appropriately prioritised.

### Nature of obligations to conduct user research

The main motivation for including user research a part of this project was that it was a requirement of the research funders. It is of note, however that although the plan for the user research proposed by the development team during the application process was extremely brief and vague, this met the funding requirements. No recommendations or guidance were provided to the developers as to what might constitute meaningful user research, nor was any evidence needed that the results of this research would be considered within the product development process.

The present research has suggested that it is not sufficient to plan to conduct user research and that this should not serve as the ‘trump card’ for funding applications. Indeed we would argue that the current simplistic requirement of some funders and regulators that users should be involved in the product development process may in fact be counterproductive. It may lead to this work becoming a box-ticking exercise, conducted to satisfy funders, rather than being a thoughtful consideration of how to conduct user research that firstly, allows the necessary information on user needs and requirements to be collected and secondly, allows the best product development decisions to be made. Taking this further, one might suggest that where user research is framed inappropriately (e.g. focusing on detailed device characteristics) that this may lead to the development path becoming more set and less amenable to change (greater lock-in) than if the user research was not done at all. It is our position that research funders should focus more on ensuring that the aims, approach, timing and integration of user research are appropriately specified and that the development decisions that are made on the basis of the research are visible.

Often the need for confidentiality leads to a reluctance to publish results and this leads to a lack of knowledge development in this area. We concur with Buckle [1] that a growing body of evidence and good-practice examples in this area would lead to a raised awareness amongst developers of how to effectively conduct and manage user research as part of a wider product development process.

Finally it is useful to reflect on what ‘good’ user needs research looks like. In this case study, in the final analysis it was not rated highly by the developer simply because the results it produced did not concur with developer expectations. In the development of technology there is a clear end point - acceptable functionality – yet the process by which this is reached remains largely invisible. In contrast, the end point of user needs research is unknown; rather research quality is a function of the process. Researchers should make developers aware that the research they conduct should be subject to evaluation and scrutiny in relation to the rigour of the process of data collection and analysis rather than on the substance of the findings *per se*.

### Recommendations

1. The guidance currently available to developers is somewhat simplistic. Funders of medical device research and development should provide developers with more detailed guidance on how to design effective user research studies. For example, what the aims of research at each stage of development should be, how it should be conducted, and what the implications of the research may be.

2. User research in the early stages of medical device development should be kept broad and aim to ‘open up’ device considerations with a focus on identifying as many clinical needs as possible. Developers should wait until a prototype is available before collecting user opinions on specific design features.

3. A formal new product process such as a stage-gate model should be adopted that allow for articulation and documentation, at pre-determined points, of the contingencies between technological development and the decisions that are made about user requirements.

4. Developers should take care when contracting out parts of development that these are objectively appraised and considered by, for example, a senior member of the development team becoming a ‘champion’ for this work.

## Conclusion

In contrast to most research in this area, this case study has provided the opportunity to explore the implementation of user needs research within a small medical device development project. In so doing we have identified numerous barriers to the implementation of user needs research that result from both formal and informal organisational processes. We have suggested the value of a formal decision making process in encouraging a process of reflection on the implications of user needs research for product development that may result in a more objective approach to decision making based on all available information. Locating funding requirements in this area would arguably lead to a more meaningful and realistic assessment of how, if at all, information about user needs should be incorporated in the product development process. Finally, the findings of this paper should be communicated by academia and also by industry bodies such as the Association of British Healthcare Industries (ABHI) in order to improve practice throughout the wider medical device industry.

## Competing interest

The authors declare that they have no competing interests.

## Authors’ contributions

JM conducted the primary data collection. JM and JB performed the data analysis, drafted the manuscript and provided domain expertise. All authors read and approved the final manuscript.

## Pre-publication history

The pre-publication history for this paper can be accessed here:

http://www.biomedcentral.com/1472-6947/12/74/prepub
